# *MYST3/CREBBP* Rearranged Acute Myeloid Leukemia after Adjuvant Chemotherapy for Breast Cancer

**DOI:** 10.1155/2014/361748

**Published:** 2014-12-08

**Authors:** Arjun Gupta, Mrinal M. Patnaik, Harris V. Naina

**Affiliations:** ^1^Department of Internal Medicine, University of Texas Southwestern Medical Center, Dallas, TX 75390, USA; ^2^Division of Hematology, Department of Medicine, Mayo Clinic, Rochester, MN 55902, USA; ^3^Department of Hematology Oncology, University of Texas Southwestern Medical Center, Seay Biomedical Building, 2201 Inwood Road, Dallas, TX 75390, USA

## Abstract

Although rare, clinicians and patients must be aware that therapy related malignancies, specifically acute myeloid leukemia (AML), can occur as a complication of adjuvant chemotherapy for breast cancer. Vigilance for signs and symptoms is appropriate. AML with t (8;16) is a specific translocation leading to formation of a fusion protein (*MYST3/CREBBP*). The *MYST3/CREBBP* AML tends to develop within 2 years of adjuvant chemotherapy, especially for breast cancer, without preceding myelodysplasia. It usually presents with disseminated intravascular coagulation and osteolytic lesions and has a poor prognosis despite aggressive resuscitation and therapy. With the increasing use of adjuvant chemotherapy for breast cancer, we are seeing a definite increase in the incidence of therapy related myelodysplastic syndromes and AML. One must keep this complication in mind while counseling and following up breast cancer patients who have received adjuvant chemotherapy. New osteolytic bone lesions in a patient with history of breast cancer do not necessarily mean metastatic disease and should be fully evaluated.

## 1. Introduction

The t(8;16)(p11.2;p13.3) is a rare translocation that has been observed in both* de novo* and therapy related acute myeloid leukemia (AML). The* MYST3/CREBBP* fusion protein results in a unique AML characterized by monocytic or myelomonocytic morphology, extramedullary involvement, disseminated intravascular coagulation (DIC), and hemophagocytosis. It has been reported in patients with breast cancer who received cytotoxic chemotherapy. Despite therapy, it generally has poor outcome. We report a case of therapy related AML with t(8;16) diagnosed 2 years after management of breast cancer in a woman who presented with DIC and osteolytic bone lesions.

## 2. Case Presentation

A 62-year-old woman presented to her local hospital with a 3-week history of low back pain associated with radicular symptoms. Two years earlier, she underwent mastectomy for an infiltrating ductal adenocarcinoma involving the left breast. The tumor formed a 5.0 × 3.5 × 2.3 cm mass with no angiolymphatic invasion and negative surgical margins. One of seven lymph nodes was positive for metastatic disease. The tumor cells were both estrogen and progesterone receptor positive (>75% staining) and HER2 negative. Following surgery, she received adjuvant chemotherapy with dose dense adriamycin and cyclophosphamide (4 cycles), followed by 4 cycles of paclitaxel. A CYP2D6 genotype was obtained and she was found to be heterozygous for the ^*^2A allele, making her an extensive metabolizer for tamoxifen. She was started on adjuvant hormonal therapy with tamoxifen and tolerated it well.

**Figure 1:**
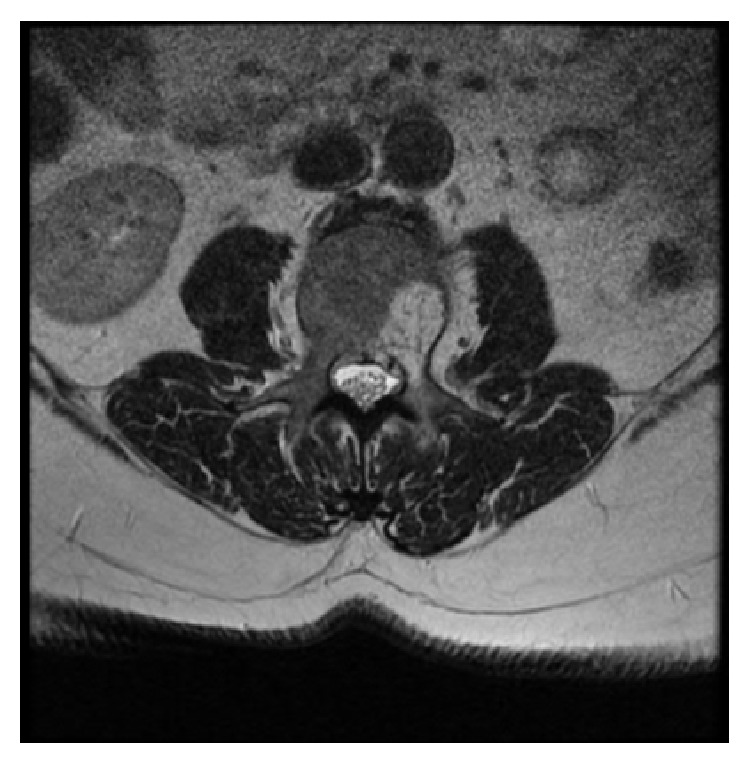
MRI of the spine; T2 weighted images demonstrating hyper intense skeletal lesions.

At her local hospital, a MRI of the spine performed due to concern for metastatic disease demonstrated diffuse lesions throughout the axial skeleton (Figure 1). The patient was noted to have a high grade fever, leukocytosis, and mucocutaneous bleeding and was referred to our institution for management.

Laboratory analysis demonstrated a leukoerythroblastic anemia (Hb 8.2 g/dL, WBC count 7.7 × 10^9^/L, 49% neutrophils, 31% lymphocytes, 4% myelocytes, 9% blasts, and 1% nucleated red cells) and disseminated intravascular coagulation (DIC) (platelet count 18,000 × 10^9^/L, prothrombin time 34 seconds, partial thromboplastin time 55 seconds, fibrinogen 120 mg/dL, and D-dimer >20,000 ng/mL with positive soluble fibrin monomer complexes).

**Figure 2:**
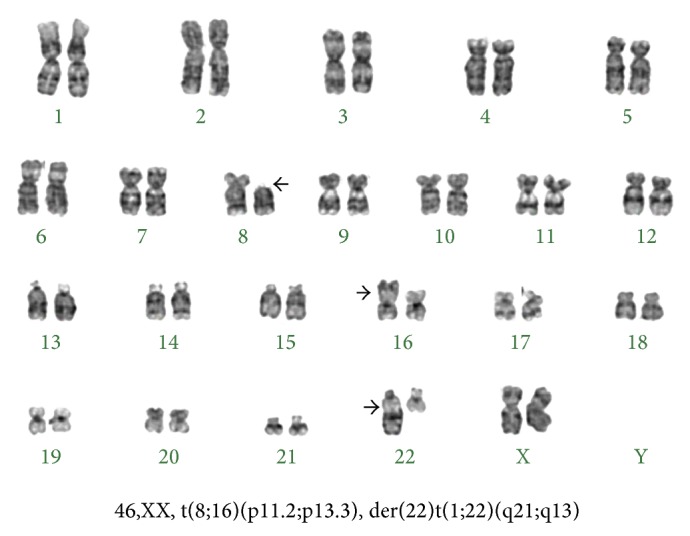
Conventional karyotype analysis of the bone marrow demonstrating t(8;16).

Differential diagnosis at this point included metastatic breast cancer versus a primary bone marrow disorder. A bone marrow aspiration and biopsy revealed increased marrow cellularity (95%) with 90% blasts. There was no evidence for myelodysplasia or hemophagocytosis. It was consistent with AML M4 (acute myelomonocytic leukemia). Cytogenetics revealed 46,XX, t(8;16)(p11.2;p13.3), der(22)t(1;22)(q21;q13) (Figure 2).

**Figure 3:**
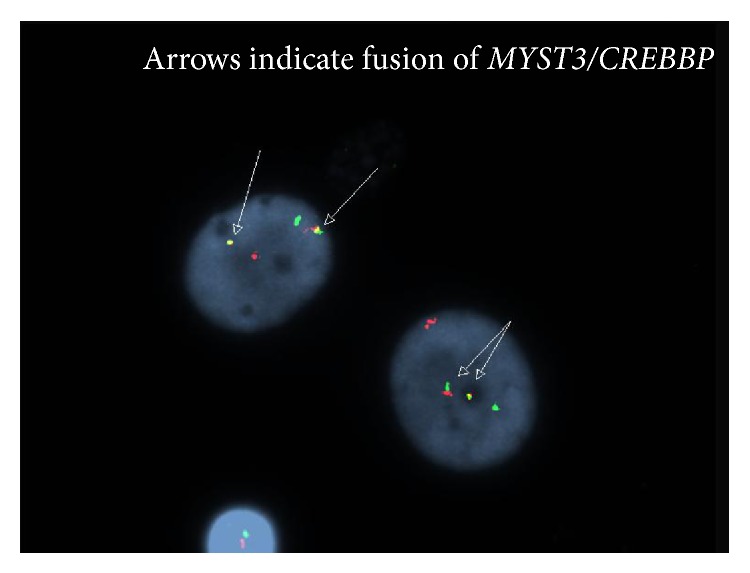
FISH analysis demonstrating the* MYST3/CREBBP* fusion. The red probe is detecting MYST3, the green probe is detecting CREBBP, and the yellow probe indicates fusion.

An AML fluorescence in situ hybridization (FISH) panel detected presence of the* MYST3/CREBBP* rearrangement (Figure 3) consistent with an 8p11.2 rearranged myeloid disorder. Along with supportive care, standard induction chemotherapy using idarubicin and cytarabine (3+7 regimen) was initiated. She unfortunately passed away on day 5 after suffering diffuse alveolar hemorrhage.

## 3. Discussion

The t(8;16)(p11.2,p13.3) is a rare translocation involved in both* de novo* and therapy related AML [1, 2]. The gene involved at 8p11.2 is* MYST3* (*MYST* histone acetyl transferase 3), previously known as* MOZ* (monocytic leukemia zinc finger [3]), and the gene involved at 16p13.3 is* CREBBP* (*CREB* binding protein) [3, 4]. The* MYST3/CREBBP* rearrangement results in a cytomorphologically unique AML characterized by monocytic or myelomonocytic morphology, extramedullary involvement, DIC, and hemophagocytosis [1, 2, 5]. Mutations in MYST4 (MORF), a paralog of MYST3, affect histone acetylation similar to MYST3 and have been specifically associated with childhood AML and therapy related myelodysplastic syndrome [6].

In one of the largest series to date, 12 of 20 females (60%) developed t(8;16) related AML, 9 to 72 months after adjuvant therapy (chemotherapy and/or radiotherapy) for breast cancer [1]. In this study seven patients had prior exposure to anthracyclines and 11 patients had prior exposure to an alkylating agent [1]. Our patient was exposed to both.

In another series, 4 of 5 female patients with* MYST3/CREBBP* related AML had received adjuvant chemotherapy with adriamycin based regimens for breast cancer [2]. In both these studies the median survival was dismal at 4.7 months [2] and 10 months, respectively [1].

Osteolytic skeletal lesions are commonly associated with plasma cell disorders but have been described in AML [7] and myeloproliferative neoplasms [8]. Our patient did present with a leukoerythroblastic blood smear, osteolytic skeletal lesions, DIC, and AML M4, two years after receiving adjuvant chemotherapy for breast cancer. Osteolytic lesions in a patient with history of breast cancer are suspicious for metastatic disease.

With the increasing use of adjuvant chemotherapy for breast cancer, we are seeing a definite increase in the incidence of therapy related myelodysplastic syndromes and AML [9, 10]. The* MYST3/CREBBP* related AML tends to develop within 2 years of adjuvant chemotherapy, without preceding myelodysplasia, and is associated with an aggressive presentation, DIC, and a poor survival. One must keep this complication in mind while counseling and following up breast cancer patients who have received adjuvant chemotherapy.
